# Sensitizing mucoepidermoid carcinomas to chemotherapy by targeted disruption of cancer stem cells

**DOI:** 10.18632/oncotarget.9884

**Published:** 2016-06-07

**Authors:** Douglas M. Guimarães, Luciana O. Almeida, Manoela D. Martins, Kristy A. Warner, Alan R. S. Silva, Pablo A. Vargas, Fabio D. Nunes, Cristiane H. Squarize, Jacques E. Nör, Rogerio M. Castilho

**Affiliations:** ^1^ Laboratory of Epithelial Biology, Department of Periodontics and Oral Medicine, University of Michigan, School of Dentistry, Ann Arbor, MI, USA; ^2^ Comprehensive Cancer Center, University of Michigan, Ann Arbor, MI, USA; ^3^ Department of Oral Pathology, School of Dentistry, University of Sao Paulo, SP, Brazil; ^4^ Department of Oral Pathology, School of Dentistry, Federal University of Rio Grande do Sul, Porto Alegre, RS, Brazil; ^5^ Department of Oral Diagnosis, Piracicaba Dental School, State University of Campinas, Campinas, SP, Brazil; ^6^ Department of Otolaryngology, Medical School, University of Michigan, Ann Arbor, MI, USA; ^7^ Department of Restorative Sciences, University of Michigan School of Dentistry, Ann Arbor, MI, USA

**Keywords:** salivary cancer, epigenetic, histone modifications, histone acetylation, cancer initiating cells

## Abstract

Mucoepidermoid carcinoma (MEC) is the most common malignancy of salivary glands. The response of MEC to chemotherapy is unpredictable, and recent advances in cancer biology suggest the involvement of cancer stem cells (CSCs) in tumor progression and chemoresistance and radioresistance phenotype. We found that histone acetyltransferase inhibitors (HDACi) were capable of disrupting CSCs in MEC. Furthermore, administration of HDACi prior to Cisplatin (two-hit approach) disrupts CSCs and sensitizes tumor cells to Cisplatin. Our findings corroborate to emerging evidence that CSCs play a key role in tumor resistance to chemotherapy, and highlights a pharmacological two-hit approach that disrupts tumor resistance to conventional therapy.

## INTRODUCTION

Malignant salivary gland tumors are uncommon lesions, representing around 3-5% of all head and neck neoplasms [1, 2]. Mucoepidermoid carcinoma (MEC) is the most common malignant salivary tumor, which comprises approximately 30% of all salivary malignances [2, 3].

Current treatment of MEC encompasses surgical resection with eventual adjuvant radiotherapy, which frequently leads to functional and aesthetic complications [3–6]. Chemotherapy is often reserved for recurrent and metastatic tumors. Administration of single-agent or combination therapy shows activity, but the overall response rates are unsatisfactory and short-lived [4, 6]. Emerging evidence suggests that the modest response of tumor cells to therapy, which results in high recurrence rates and poor survival, are associated with the presence of cancer stem cells (CSCs) [7, 8]. Indeed, the presence of CSCs is considered as a prognosis determinant in several cancers, including those of the ovary, lung, breast and head and neck [9–12]. Resistance of tumors to chemotherapy is associated with the gain of new mutations, activation of specific signaling pathways, the presence of CSCs, and histone modifications as epigenetic modulators of cancer behavior [12–16]. Interestingly, histone modifications may play a major role in the establishment of CSCs and tumor cells that are resistant to chemotherapy [17]. By dynamically modulating tumor chromatin folding, histone deacetylation leads to reduced transcription of differentiation genes and activation of stem cell genes. These results in the maintenance of tumor cells in a quiescent stage, making them more resistant to conventional intercalating agents compared to non-quiescent tumor cells [18, 19]. In fact, changes in DNA folding induce various cellular phenotypes mediated by cell type-specific chromatin organization [20]. We have recently shown that pharmacological acetylation of chromatin in head and neck squamous cell carcinoma (HNSCC) results in dramatic changes on cellular phenotype [21]. We also found that acetylation of tumor histones by histone deacetylase inhibitors (HDACi) abrogates tumor resistance of HNSCC to chemotherapy [12]. Indeed, similar findings have been reported in other tumors including non-small cell lung cancers, osteosarcomas, mesotheliomas, and cervical cancer [22–25]. Further, HDACi disrupts tumorspheres, suggesting a direct role of chromatin organization in the maintenance of CSCs [12].

Recent advances in MEC biology have occurred through the identification of a subpopulation of CSCs with high tumorigenic properties [26]. However, the role of CSCs in chemoresistance in MEC is poorly understood. We investigated the effects of HDACi and Cisplatin in CSCs derived from two MEC cell lines and found that CSCs did not respond to Cisplatin. In fact, Cisplatin triggered the accumulation of CSCs in one of the MEC tumor cell lines. We next examined whether HDACi had any effects over the population of CSCs and found that an extremely low concentration of HDACi is sufficient to deplete MEC CSCs. Further, sensitization of tumor cells with HDACi prior to treatment with Cisplatin depleted MEC CSCs, surpassing the inhibitory effects of HDACi alone. In addition to improving the therapeutic effects of Cisplatin, HDACi also reduces the half dose inhibitory concentration (IC_50_) of Cisplatin in MEC tumor cells.

Our findings suggest that treating MEC with drugs that induces chromatin acetylation result in the destruction of CSCs and reduces chemoresistance of MEC cells.

## RESULTS

### MEC have mixed levels of histone H3 acetylation

**Figure 1:**
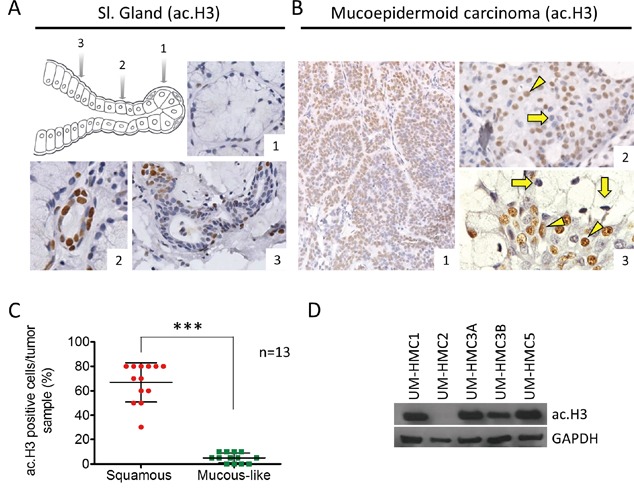
Levels of ac. Histone H3 in salivary glands and MEC **A.** Representative expression of ac. histone H3 in acinar cells (A_#1), intercalated duct (A_#2) and secretory ducts (A_#3) of salivary glands. **B.** MEC has mixed levels of ac. histone H3, with epidermoid and intermediate cells showing both positive and negative staining; in addition, mucous-like tumor cells negative for ac. histone H3 are found next to positive epidermoid tumor cells. **C.** Quantification of ac. histone H3 using tumor tissue reveals increased acetylation in squamous and intermediate tumor cells (red) compared to mucous-like tumor cells (green). **D.** Western blot analysis of baseline expression of ac. histone H3 in UM-HMC1, UM-HMC2, UM-HMC3A, UM-HMC3B, and UM-HMC5 tumor cells. GAPDH served as a loading control.

The acetylation status of histones is controlled by the balance between histone deacetylase (HDAC) and histone acetyltransferase (HAT) activity. Changes in histone acetylation have a direct effect on many cellular functions, including cellular morphology and response to environmental cues [21, 27–29]. Interestingly, we found that normal human salivary glands have different expression patterns of histone H3 (Lys9) within different cell populations. We found that acinar cells (Figure 1A_#1) were hypoacetylated compared to epithelial cells from the intercalated (Figure 1A_#2) and secretory (Figure 1A_#3) ducts. Similar to normal salivary glands, MEC were comprised of a heterogeneous population of epithelial cells in distinct stages of cellular differentiation and histone H3 (Lys9) acetylation (Figure 1B_#1). Tumor cells showing squamous and intermediate differentiation had high levels of histone H3 acetylation (mean: 66.92±4.441) (Figure 1B_#2 arrowhead), but a smaller number of tumors cells were negative for histone H3 (Figure 1B_#2 arrow). Mucous-like tumor cells were primarily negative for histone H3 (Mean: 5±1.132) (Figure 1B_#3 arrow) compared to differentiated squamous cells (Figure 1B_#3 arrowhead). Quantification revealed that histone H3 was significantly acetylated in squamous and intermediate differentiated cells compared to mucous-like tumor cells from thirteen cases of MEC (*** p<0.001) (Figure 1C).

Tumor cell lines derived from primary and metastatic MEC were established in the Nör laboratory at the University of Michigan [26, 30]. We examined histone H3 acetylation in 5 MEC cell lines, including UM-HMC1, UM-HMC2, UM-HMC3A, UM-HMC3B, and UM-HMC5. With the exception of UM-HMC2, the other 4 MEC cell lines had detectable histone H3 acetylation (Figure 1D). As previously reported, MEC cell lines generate tumors following engraftment onto SCID mice [30]. Interestingly, in addition to generating viable xenografts, MEC cell lines recapitulate many of the features of the primary tumors. In general, solid tumors generated from engrafted MEC cell lines were primarily comprised of squamous-like tumor cells and patches of mucous-like tumor cells (Supplementary Figure 1A). Tumor cell lines derived from xenografts also retained cytokeratin 5 expression suggesting the presence of tumor cells with epidermoid differentiation, along Vimentin expression, which are commonly observed in MEC (Supplementary Figure 1B) [31, 32].

Collectively, these findings strongly suggest that MEC cell lines retain multipotent ability to differentiate into squamous and mucous-like cellular components. Nonetheless, these cell lines differ in chromatin acetylation (histone H3 lys9 acetylation), suggesting they will display differential gene transcription patterns and distinct cellular behavior.

### MEC cell lines generate tumorspheres

Solid tumors are often refractory to current therapies, and this is the case with MEC [4–6]. New findings suggest that CSCs are involved in the development of chemoresistance and radioresistance [7, 8, 33]. These observations have sparked interest in developing therapeutic strategies targeting CSC function and fate. Growing tumor cells in ultra-low adhesion conditions generates tumor spheres of different sizes and shapes [34]. We have recently demonstrated that the generation of tumor spheres is a useful technique for enriching CSCs *in vitro* and is of interest for developing new therapies targeting CSCs [35]. Epithelial spheres advent from normal epithelial cells and tumor spheres are classified into three groups, including 1) holospheres, which are characterized by the formation of a large circular sphere with regular borderlines; 2) paraspheres, which are small sphere-like structures with fragmented borderlines; and 3) merospheres, which form sphere-like structures with intermediated morphology between holospheres and paraspheres [34, 35]. Different populations of head and neck CSCs grown under ultra-low adhesion are characterized by distinct biological behavior and CSC content. While holospheres present low proliferation index, it can accumulate more ALDH positive cells compared to mero and paraspheres and efficiently invade a reconstituted basal membrane layer. Holospheres is also characterized by an improved ability to adhere to substrates and to retain the ability to form holo, mero, and paraspheres upon serial passages [35]. Most interesting, tumor cells have also shown to retain its stem cell hierarchies upon serial cellular passages that suggest the maintenance of an asymmetrical cellular division. Of interest, serial passages also enhanced stem cell self-renewal [36].

**Figure 2:**
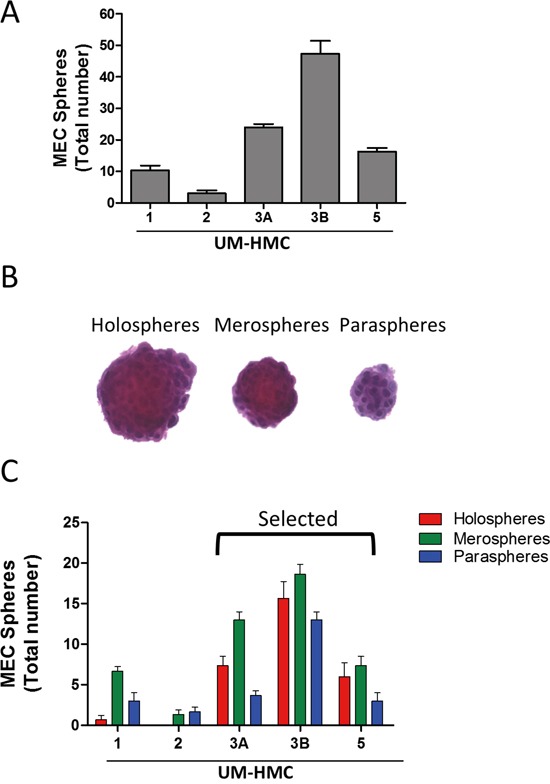
MEC cell lines generate tumor spheres **A.** All MEC cell lines generate tumor spheres when grown in ultra-low adhesion conditions. **B.** Representative tumor spheres from MEC cells showing holosphere-, merosphere-, and parasphere-like shapes. **C.** Quantification of the total number of holospheres, merospheres, and paraspheres individually produced by UM-HMC1, UM-HMC2, UM-HMC3A, UM-HMC3B, and UM-HMC5 cell lines.

We found that all MEC cell lines generated tumor spheres when grown under ultra-low adhesion conditions (Figure 2A). Interestingly, the MEC cell lines showed differential efficiency in forming tumor spheres. UM-HMC2 cells had low efficiency in generating tumor spheres, with a mean of 3 spheres per 2,500 MEC cells compared to metastatic UM-HMC3B cells, which produced a mean of 47.33 tumor spheres per 2,500 MEC cells. UM-HMC1, UM-HMC3A, and UM-HMC5 generated an intermediate number of tumor spheres (means of 10.33, 24, and 16.33, respectively). Tumor colonies and spheres are also generated by other malignant tumor cell lines, including those of the pancreas [37], breast [9], prostate [38], colon [39], head and neck [36], and, most recently, MEC [40, 41].

Interestingly, with the exception of UM-HMC2 cells, all other MEC cells generated all three subtypes of tumor spheres (Figure 2B). However, each cell line was unique in the number and type of spheres that were formed (Figure 2C). While UM-HMC1 and UM-HMC2 cells had a limited potential to generate viable spheres, UM-HMC3A, UM-HMC3B, and UM-HMC5 cells generated a larger number of tumor spheres (Figure 2C). In addition, the proportion of holospheres, merospheres, and paraspheres produced by UM-HMC3A, UM-HMC3B, and UM-HMC5 cells were similar, but meroclones accounted for the majority of tumor spheres followed by holospheres and then paraclones (Figure 2C).

Because UM-HMC3A, UM-HMC3B, and UM-HMC5 cells yielded the highest number of tumor spheres, we used these cells for our remaining experiments. The presence of CSCs in UM-HMC3A, UM-HMC3B, and UM-HMC5 cells was further confirmed by detection of aldehyde dehydrogenase (ALDH), a well-known biomarker for various normal and cancer stem cells (Supplementary Figure 2) [42–46].

### Cisplatin differentially affects the population of CSCs in MEC cell lines

**Figure 3:**
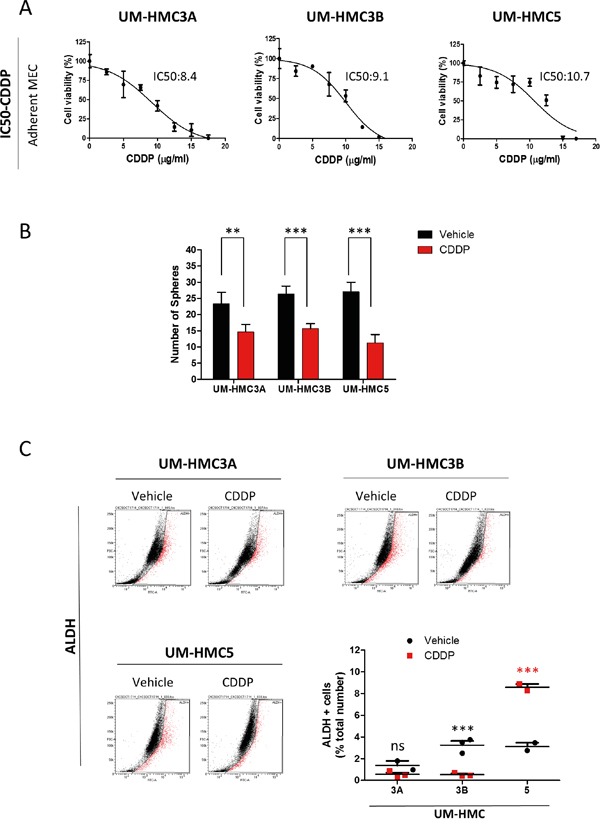
MEC tumor cell lines respond differentially to Cisplatin **A.** Determination of the IC_50_ of Cisplatin in MEC cells. **B.** Cisplatin reduces the number of tumor spheres in UM-HMC3A, UM-HMC3B, and UM-HMC5 (**p<0.005; ***p<0.001). **C.** ALDH activity following administration of Cisplatin. ALDH-positive UM-HMC3A cells do not respond to Cisplatin, while UM-HMC3B are highly sensitive to Cisplatin. Cisplatin increases the number of ALDH-positive tumor cells in UM-HMC5.

We next examined the effects of Cisplatin on CSCs. We first determined the IC_50_ of Cisplatin in each MEC cell line. UM-HMC3A had an IC_50_ of 8.47 μg/ml, which was lower than UM-HMC3B with an IC_50_ of 9.17 μg/ml and UM-HMC5 with an IC_50_ of 10.7 μg/ml (Figure 3A). We then treated MEC cells with Cisplatin at the appropriate IC_50_ concentrations and performed a sphere forming assay to determine the effects of Cisplatin on tumor cells enriched for CSC (Figure 3B). Cisplatin alone sufficiently reduced the number of viable tumor spheres in all three MEC cell lines (UM-HMC3A **p<0.005; ***p<0.001 for UM-HMC3B and 5). Interestingly, when using ALDH^+^ tumor cells to assess the effect of Cisplatin on CSCs, we observed that each MEC cell line reacted differently (Figure 3C). Although CSCs derived from UM-HMC3A did not respond to Cisplatin (ns p>0.05), UM-HMC3B CSCs showed high sensitivity to Cisplatin (*** p<0.001). The number of ALDH^+^ cells in UM-HMC5 CSCs increased from 4.1% in response to vehicle to 6.8% in response to Cisplatin (*** p<0.001). The discrepancy between the tumorsphere forming assay and the total number of ALDH^+^ cells prompted us to determine whether the subtypes of tumorspheres would respond differently to chemotherapy, as we have previously observed in HNSCC, and if differential responses would be due to CSCs with distinct behavior and ability to respond to chemotherapy. Indeed, we found that holospheres were more sensitive to Cisplatin compared to merospheres and paraspheres (Supplementary Figure 3A, 3B, 3C). Remarkably, paraspheres were completely refractory to therapy in two MEC cell lines (UM-HMC3A and 3B) (Supplementary Figure 3A and 3B). Similarly, paraspheres derived from UM-HMC5 showed less response to Cisplatin compared to holospheres and merospheres (Supplementary Figure 3C).

These unexpected results suggest that we can better understand the biology of tumor cells during chemotherapy through the combination of ALDH enzymatic detection and the enrichment of CSCs using an ultra-low adhesion culture technique. While the total number of tumorspheres suggests that MEC CSCs are sensitive to cisplatin, ALDH expression suggests that they are either non-responsive or proliferate in the response to cisplatin. Moreover, classification of tumorspheres into holospheres, merospheres, and paraspheres helps to identify which MEC tumors cells will be resistance to cisplatin (paraspheres). Indeed, our findings corroborate with the observations in MEC patients in which single-agent or combination therapy is effective for short periods of time, suggesting that tumor rebound may be associated with the accumulation of CSC [4, 6].

### SAHA efficiently reduces CSCs in MEC cell lines

**Figure 4:**
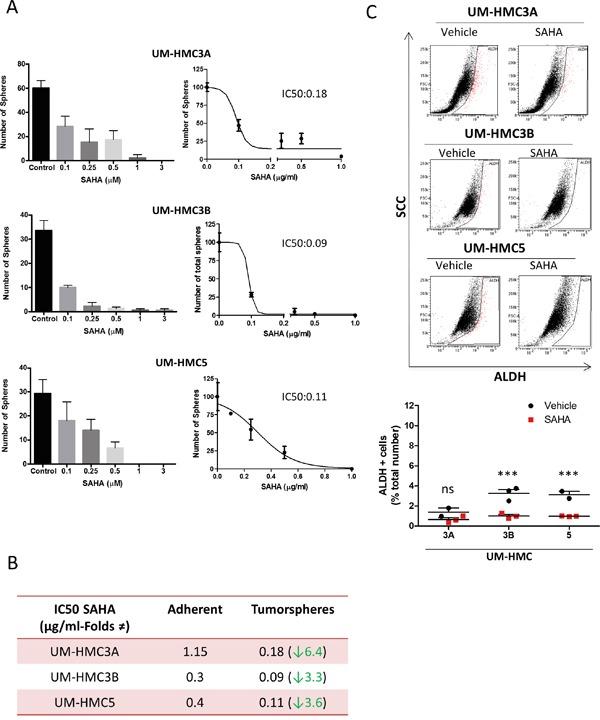
SAHA reduces CSCs in MEC cell lines **A.** Determination of IC_50_ of SAHA in tumor spheres derived from UM-HMC3A, UM-HMC3B, and UM-HMC5 cell lines. **B.** Tumor cells derived from tumor spheres show increased sensitivity to SAHA compared to tumor cells growing in a monolayer. **C.** Low levels of SAHA (IC_50_ for tumor spheres) reduce the number of ALDH-positive tumor cells. Notably, UM-HMC3A did not respond to SAHA, while CSCs were significantly reduced in UM-HMC3B and UM-HMC5 (*** p<0.001).

The HDACi SAHA (Vorinostat) has been used in several types of tumors [47, 48]. SAHA targets histone and nonhistones substrates by removing the acetyl moiety from the lysine residues of proteins that include core histones. SAHA act as an inhibitor of classes I, and II of histone deacetylases inhibiting the proliferation of tumor cells [49, 50], inhibition of mitosis [51, 52], cell cycle arrest [53] differentiation or apoptosis [54] with little toxicity to normal cells. Administration of SAHA induces the acetylation of tumor chromatin as detected by ac.H3 (Lys9) (Supplementary Figure 4A). Acetylation of core histone proteins also drives cellular differentiation while restricting transformation [55, 56]. We assessed the IC_50_ of SAHA in adherent MEC cell lines. UM-HMC3A were very resistant to SAHA (1.15 μM) compared to UM-HMC5 (0.4 μM) and UM-HMC3B (0.3 μM) (Supplementary Figure 4B). Additionally, we examined the inhibitory effects of SAHA in CSCs. To accomplish this, we enriched for CSCs by seeding MEC cells in ultra-low adhesion conditions to generate tumor spheres and determined the IC_50_ for the tumor spheres produced by each cell line (Figure 4A). Administration of SAHA alone was able to efficiently disrupted the ability of MEC cells to generate tumor spheres in a dose-dependent manner (Figure 4A). Upon analysis of all subpopulation of spheres composed by holo, mero and paraspheres we observed that the effect of SAHA was homogeneous throughout all tumor spheres in a dose-dependent way (Supplementary Figure 4C).

Tumor spheres grown in ultra-low attachment conditions were more sensitive to SAHA than tumor cells grown in monolayers (UM-HMC3A_6.4 folds reduction, UM-HMC3B_3.3 folds reduction, and UM-HMC5_3.6 folds reduction (Figure 4B).

We next examined the effects of SAHA on ALDH^+^ CSCs. The number of ALDH^+^ cells was reduced following treatment with SAHA at the concentrations used in tumor spheres (Figure 4C). Unlike CSCs from UM-HMC3A (ns p>0.05), the reduction in CSCs from UM-HMC3B and UM-HMC5 was significant (*** p<0.001). The resistance of UM-HMC3A to SAHA (Supplementary Figure 4B) may explain why we did not see statistically significant reductions in CSCs from this cell line.

Unlike Cisplatin, SAHA did not increase the number of ALDH^+^ cells or the accumulation of paraspheres.

### Sequential administration of SAHA and Cisplatin (two-hit approach) reduces the number of CSCs, prevents resistance in MEC, and reduces the amount of Cisplatin required to achieve IC_50_ levels

**Figure 5:**
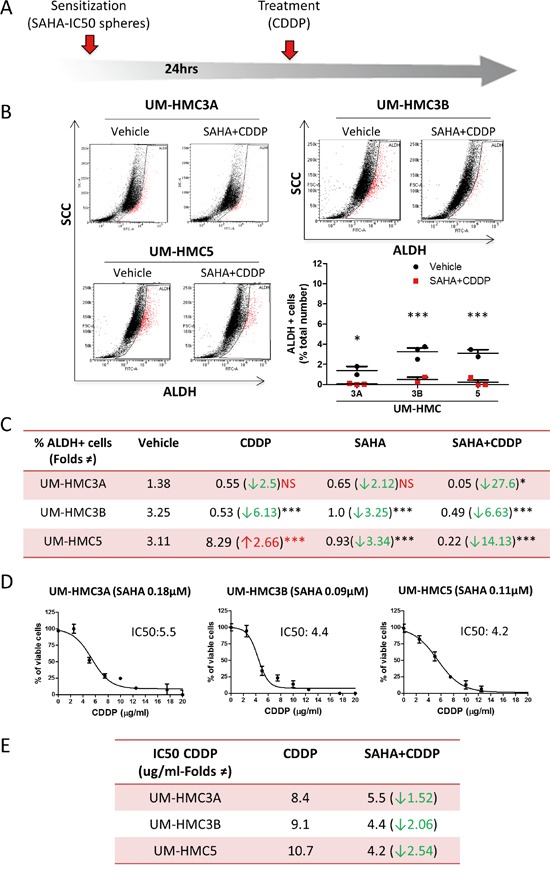
SAHA sensitizes tumor cells to Cisplatin **A.** Representation of the “two hit approach” using SAHA 24hrs prior to Cisplatin to sensitize tumor cells. **B.** The two-hit approach significantly reduces ALDH-positive cells (*p<0.05; ***p<0.001). **C.** The table depicts the efficiency of SAHA in sensitizing all MEC tumor stem cells to Cisplatin compared to the administration of Cisplatin or SAHA alone. **D.** Graphic determination of the new IC_50_ of Cisplatin after sensitization with SAHA. **E.** Note the reduction in the IC_50_ of Cisplatin is 1.52 fold in UM-HMC3A, 2.06 fold in UM-HMC3B, and 2.54 fold in UM-HMC5 upon sensitization with SAHA compared to Cisplatin alone.

Previous studies, including from our group, have shown that SAHA increases the sensitivity of solid tumors to Cisplatin [12, 57–60]. Resistance to chemotherapy is a complex and poorly understood process that results from many factors, including activation of resistance pathways, such as NFκB signaling, and the presence of CSCs [12, 21]. Because different mechanisms underlie resistance, solid tumors often show a mixed response to therapy. We showed that CSCs from MEC cell lines respond differently to Cisplatin. We have previously found that histone deacetylation disrupts HNSCC tumor spheres [12, 21]. Our goal was to deliver a two-hit therapy that would sensitize CSCs to Cisplatin by first inducing histone deacetylation (SAHA) (Figure 5A). This approach resulted in a homogeneous response of all MEC tumor cell lines to Cisplatin, with significant reductions in CSCs, as determined by ALDH levels (Figure 5B) and a reduction in tumorspheres (Supplementary Figure 5); these effects were not observed following treatment with Cisplatin or SAHA alone (Figure 5C).

We next examined whether the reduction in CSCs translated into the sensitization of MEC tumor cells to Cisplatin. MEC cells were sensitized with SAHA for 24 hours followed by administration of Cisplatin (Figure 5D). SAHA sensitized MEC cells to Cisplatin, resulting in a reduction of the IC_50_ from 8.4 μg/ml to 5.5 μg/ml for UM-HMC3A, from 9.1 μg/ml to 4.4 μg/ml for UM-HMC3B, and from 10.7 μg/ml to 4.2 μg/ml for UM-HMC5 (Figure 5E). In addition to suggesting that the two-hit therapy is more efficient in ablating CSCs, our data also shows that this strategy reduces the amount of Cisplatin required to achieve IC_50_ levels.

## DISCUSSION

The treatment for salivary MEC includes surgical resection and eventual adjuvant radiotherapy because this disease is resistant to conventional chemotherapy [3–6]. Recent studies suggest that CSCs play a major role in tumor relapse and treatment failure [7, 33, 47]. We found that all MEC cells lines contain CSCs that form spheres when cultured in ultra-low adhesion conditions. In addition, MEC cell lines contain different numbers of CSCs, distinct abilities to generate tumor spheres, and differential capacities to form the various subtypes of tumor spheres. We further observed that MEC have heterogeneous H3 acetylation patterns. For example, tumors cells with squamous and intermediate differentiation are commonly acetylated, but hypoacetylated tumor cells are also present within the tumor mass. Interestingly, mucous-like tumor cells are also hypoacetylated. Indeed, the level of histone acetylation correlates with the transcriptional activity of the cell [61, 62]. Histone acetylation is associated with increased transcription of genes involved in differentiation, and histone deacetylation is associated with reduced gene transcription and/or activation of stem cell-associated genes. Changes in the levels of histone acetylation has been observed in various processes, including the initiation and progression of cancer, cellular plasticity, inflammation, maintenance of CSCs, and activation of tumor resistance pathways [12, 21, 56]. We have observed that certain head and neck tumor cell lines are prone to histone deacetylation upon administration of chemotherapy [12]. Moreover, these cells are characterized by increased resistance to chemotherapy. Given that histone deacetylases are involved in transcriptional repression, we hypothesize that histone deacetylation in tumor cells selectively activates stem cell-associated genes, resulting in chemoresistance. If true, pharmacological inhibition of histone deacetylases in all tumors cells would disrupt signaling programs associated with maintenance of CSCs, thereby reducing the resistance of MEC to Cisplatin. Evidence of the efficacy of such combined therapy has been demonstrated in Hodgkin's Lymphoma, cervical cancer, ovarian cancer, head and neck squamous cell carcinoma, and pancreatic cancer [12, 57–60, 63]; however, the mechanism of action remain elusive. Our work focuses on the response of CSCs to chemotherapy. Interestingly, we found that CSCs derived from MEC cell lines show variable responses to Cisplatin. While CSCs derived from UM-HMC3A did not respond to Cisplatin, CSCs derived from a metastatic MEC (UM-HMC3B) were sensitive to this drug. Perhaps the most interesting result came from UM-HNC5 cells, in which Cisplatin induced an abrupt accumulation of CSCs. Although unexpected, CSCs in other tumors accumulate in response to chemotherapy [64–66]. Unlike Cisplatin, SAHA reduced the number of tumor spheres and ALDH^+^ cells in all tumor cell lines. These results suggest that pharmacological acetylation of tumor cells has a direct impact on the viability of CSCs. Indeed, HDACi sensitizes tumor cells to chemotherapy and radiotherapy [22–25]. Although poorly understood, HDACi-induced decondensation of chromatin makes DNA more permeable to chemotherapeutic agents [12, 58]. In lung cancer, HDACi increases apoptosis of tumor cells through a Bax-dependent mechanism [67]. As a molecular target of HDACi, increased acetylation of Ku-70 and a reduction in its DNA-binding affinity occur in prostate cancer [68].

Several other mechanisms have been associated with chemotherapy resistance of CSCs. Among them, drug efflux play a role in tumor resistance to chemotherapy by pumping out anticancer drugs from cancer cells. Recent findings have shown that glioma CSCs present high drug efflux capability in a mechanism that involves the activation of ABC transporters [69]. Such enhanced drug efflux was also observed in hepatocellular carcinomas (HCC) CSCs in a mechanism dependent on the activation of Akt [70]. Remarkably, a key component of anticancer drug resistance observed on CSCs is the expression of high levels of ALDH [71–74]. Targeted inhibition of ALDH activity results in reduced chemoresistance of gastric, lung cancer and leukemic cells [71, 75]. Altogether, high levels of ALDH seems to be required to maintain the population of CSCs and to confer resistance of CSCs to cancer therapy. Interestingly, the expression of Notch gene, previously associated with the maintenance of stem cells [76], acts as an upstream regulator of ALDH activity [77]. Notch itself is negatively regulated by histone acetyltransferases like Tip60 through acetylation [78]. These evidences align with our findings in which histone acetylation (mediated by the histone deacetylase inhibitor SAHA) result in a reduction of ALDH + cells leading to an enhanced sensitivity of MEC cells to Cisplatin.

The efficiency in reducing the number of CSCs through administration of SAHA suggests that our two-hit therapeutic strategy is effective, as evidenced by a 27-fold reduction in ALDH^+^ cells. In contrast to their combination, SAHA or Cisplatin alone did not efficiently reduce CSCs.

SAHA was also effective in reducing the concentration of Cisplatin needed to achieve IC_50_. This result is of particular interest for patients that fail their initial chemotherapy cycles due to high toxicity [79].

In summary, we presented evidence that administration of HDACi efficiently disrupts MEC-containing CSCs and reduces the concentration of Cisplatin required to achieve IC_50_. Our research helps to clarify the mechanism of action of HDACi and highlights an efficient therapeutic strategy for managing MEC. Future in vivo studies are required to confirm the feasibility of this strategy.

## MATERIALS AND METHODS

### Tissue microarray (TMA)

TMAs were constructed using tissue samples from thirteen mucoepidermoid carcinomas, salivary gland tumors and control normal salivary gland tissues using a manual tissue arrayer (Sakura Co, Japan). Three representative cylindrical cores of 2.0 mm diameter were removed from each tissue block and arranged sequentially in a ready-to-use paraffin block (Sakura Co, Japan), according to Fonseca et al [80].

### Immunohistochemistry and immunofluorescence

For immunohistochemical staining, the slides were incubated overnight with anti-acetyl histone H3 (Cell Signaling, Danvers, MA, USA) and then anti-rabbit secondary antibody for 60 minutes at RT (Vector laboratories, Burlingame, CA, USA). HRP was detected using the Vector DAB detection system, and the slides were counterstained with Mayer's hematoxylin. For immunofluorescence assays, the samples were fixed with methanol at −20°C for 6 minutes, blocked with 0.5% (v/v) Triton X-100 in PBS and 3% (w/v) bovine serum albumin (BSA) and incubated with anti-Vimentin (Thermo Scientific, Waltham, MA, USA), anti-BMI-1 (Millipore, Billerica, MA, USA), anti-Pan-cytokeratin (Cell Signaling, Danvers, MA, USA), anti-phospho S6 (Cell Signaling, Danvers, MA, USA) and ac.H3 (Lys9) (Cell Signaling, Danvers, MA, USA). Cells were then incubated with FITC- or TRITC-conjugated secondary antibody and stained with Hoechst 33342 (Sigma-Aldrich Corp., St. Louis, MO, USA) to visualize DNA content. Images were taken using a QImaging ExiAcqua monochrome digital camera attached to a Nikon Eclipse 80i Microscope (Nikon, Melville, NY) and visualized with QCapturePro software.

### Cell lines

Mucoepidermoid carcinoma cells lines UM-HMC1, UM-HMC-2, UM-HMC-3A, UM-HMC-3B and UM-HMC5 were originally established at the University of Michigan School of Dentistry and described by Warner et al (2013). Cells lines were maintained in a 5% CO_2_ humidified incubator at 37°C and cultured in RPMI 1640 (Thermo Scientifics, Waltham, MA, USA) supplemented with 10% fetal bovine serum (Thermo Scientifics), 1% antibiotic (Invitrogen, Carlsbad, CA, USA), 1% L-glutamine (Invitrogen), 20 ng/ml epidermal growth factor (Sigma–Aldrich, St. Louis, MO, USA), 400 ng/ml hydrocortisone (Sigma–Aldrich), and 5 μg/ml insulin (Sigma–Aldrich). Cells were treated with SAHA (Cayman Chemical Company Ann Arbor, MI, USA) and Cisplatin (Sigma-Aldrich, St. Louis, MO, USA).

### MEC xenograft-derived tumor tissue samples

UM-HMC-derived xenograft tumors were established in the Nör laboratory at the University of Michigan. Briefly, tumors were surgically retrieved when they reached 800–1000 mm^3^, fixed overnight in 10% buffered formalin (Fisher) at 4°C, and paraffin embedded [30]. Unstained slides were sectioned at 3 μm thickness and processed for hematoxylin and eosin (H&E) staining.

### IC_50_ determination

We used the CellTiter 96TM AQueous non-radioactive cell proliferation kit (Promega) to determine concentrations of Cisplatin and SAHA that inhibited cell proliferation by 50% (IC_50_). Cell proliferation was determined by reduction of MTS (3-(4,5-dimethylthiazol-2-yl)-5-(3-carboxymethoxy phenyl)-2-(4-sulfophenyl)-2H-tetrazolium, inner salt) following the manufacturer's protocol. In brief, 2500 cells were plated into 96-well plates and treated in triplicate with control (vehicle), SAHA (0.1, 0.25, 0.5, 1.0, 2.5, 5.0 and 10.0 μM), or Cisplatin (2.5, 5.0, 7.5, 10.0, 12.5, 15.0, 17.5 and 20.0 μg/ml) for 24 h. Cells were incubated with MTS at 37°C for 4h, and the results were assessed by absorbance (Bio-Tek EL-311, Bio-Tek Instruments) at 490 nm.

### Tumor sphere formation assay

MEC cells were plated on ultra-low attachment plates and grown for 5 days. Sphere formation was observed daily. SAHA was administered on the first day of culture to evaluate sphere formation in the presence of histone H3 acetylation. Cisplatin was added on day 5 of culture for 24 hours. Spheres growing in suspension were collected at day 6, transferred to a glass slide by centrifugation (1500 rpm, 4°C, 10 minutes) using a cytospin system, and fixed with PFA for 15 minutes at RT.

### Flow cytometry

CSCs derived from MEC tumors were identified by flow cytometry for ALDH (aldehyde dehydrogenase) activity using the Aldefluor kit (StemCell Technologies, Durham, NC, USA) according to the manufacturer's instructions. Briefly, UM-HMC3, UM-HMC3B, and UM-HMC5 cells were treated with SAHA and/or Cisplatin at concentrations determined by the IC_50_ studies. Cells were then suspended with uncharged Aldefluor substrate BAAA (BODIPY-aminoacetaldehyde) that is converted intracellularly into negative charged BAA- by endogenous ALDH resulting in the production of bright fluorescence. The Aldefluor substrate BAAA (BODIPY-aminoacetaldehyde), and negative control (diethylaminobenzaldehyde, a specific ALDH inhibitor) was incubated for 45 minutes (optimized for MEC cells) at 37°C. All samples were analyzed in a FACS Canto IV (BD Biosciences, Mountain View, CA, USA, 520-540nm) at the University of Michigan Flow Cytometry Core. Gates were determined using DEAB control. ALDH profile was determined in MEC cells upon incubation of Aldefluor for 45 minutes. ALDH bright tumor cells were determine by an overlay of DEAB treated cells and ALDH bright cells. Briefly, tumor cells were initially gated using a forward scatter (FSC) and side scatter (SSC) dot plot to eliminate debris. The fluorescence channel is initially setup using DEAB treated cells area (low bright or negative cells). The percentage of ALDH bright cells was gated outside of DEAB treated cells area (R2 or ALDH). Each tumor cell line was analyzed using its own DEAB x SCC settings to account for differences in the cell surface and complexity.

### Immunoblotting

Cells were harvested in RIPA buffer and briefly sonicated. Protein lysates (30 μg) were separated by 10% to 15% SDS–PAGE and transferred to a polyvinyl difluoride membrane (Immobilon) (Millipore). Membranes were blocked in 0.1 M Tris (pH 7.5), 0.9% NaCl and 0.05% Tween-20 (TBS-T) with 5% nonfat dry milk. Membranes were incubated with anti-acethyl-Histone H3 (Lys9) (Cell Signaling). GAPDH (Calbiochem) served as a loading control. The reaction was visualized using ECL SuperSignal West Pico Substrate (Pierce Biotechnology).

### Statistical analysis

All statistical analysis was performed using GraphPad Prism (GraphPad Software, San Diego, CA). Statistical analysis of total number of squamous and mucous-like tumor cells positive to ac.H3 was performed using t-test. Statistical analysis of total number of tumor spheres, and the % of tumor cells positive for ALDH receiving CDDP alone, SAHA alone, and the combination of SAHA and CDDP was performed using two-way analysis of variance (ANOVA) followed by Bonferroni posttest. Percentage of UM-HMC cells presenting acetylated upon SAHA treatment was analyzed using one-way ANOVA followed by Tukey's Multiple Comparison Test. Asterisks denote statistical significance (* p < 0.05; ** p < 0.01; *** p < 0.001; and NS p > 0.05). All samples were normalized to 100% following nonlinear regression to fit the data to the log(inhibitor) vs. response (variable slope) curve.

## SUPPLEMENTARY FIGURES


