# Supplementary material for: Sensitizing mucoepidermoid carcinomas to chemotherapy by targeted disruption of cancer stem cells
Source: Oncotarget. 2016 Jun 7;7(27):42447–60. doi: 10.18632/oncotarget.9884 (PMC5173147; doi:10.18632/oncotarget.9884)
Supplement: Supplementary file 1 [file oncotarget-07-42447-s001.pdf]

Sensitizing mucoepidermoid carcinomas to chemotherapy by targeted disruption of cancer stem cells

SUPPLEMENTARY FIGURES

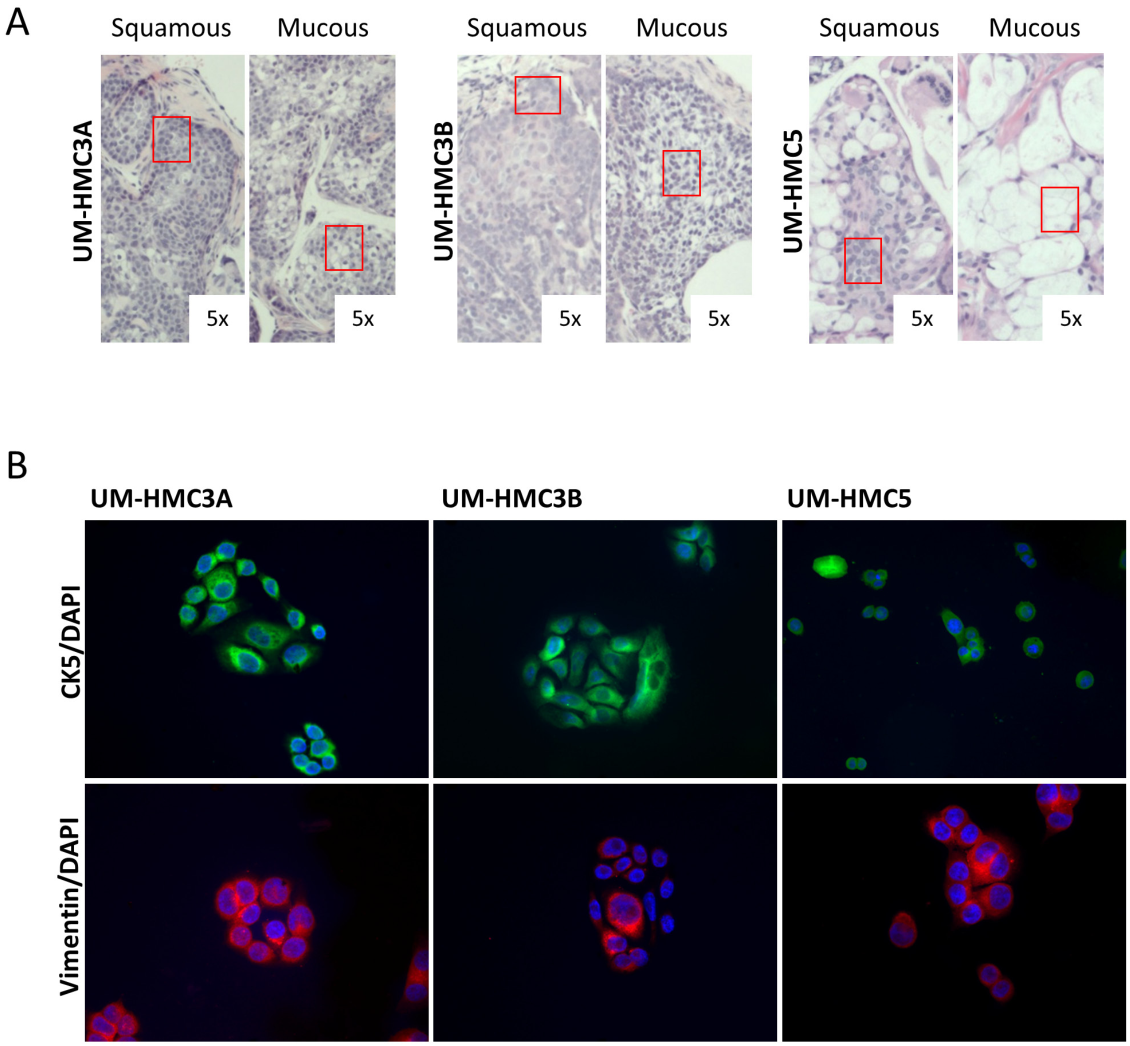

Supplementary Figure S1: MEC cell lines retain histological features of human MEC tumors. A. H&E sections from UM-HMC3A, UM-HMC3B and UM-HMC5 xenograft tumors shows the presence of squamous-like and mucous-like cells. **B.** UM-HMC3A, UM-HMC3B and UM-HMC5 are positive for the epithelial cell marker cytokeratin (CK) 5 (green) and vimentin (red).

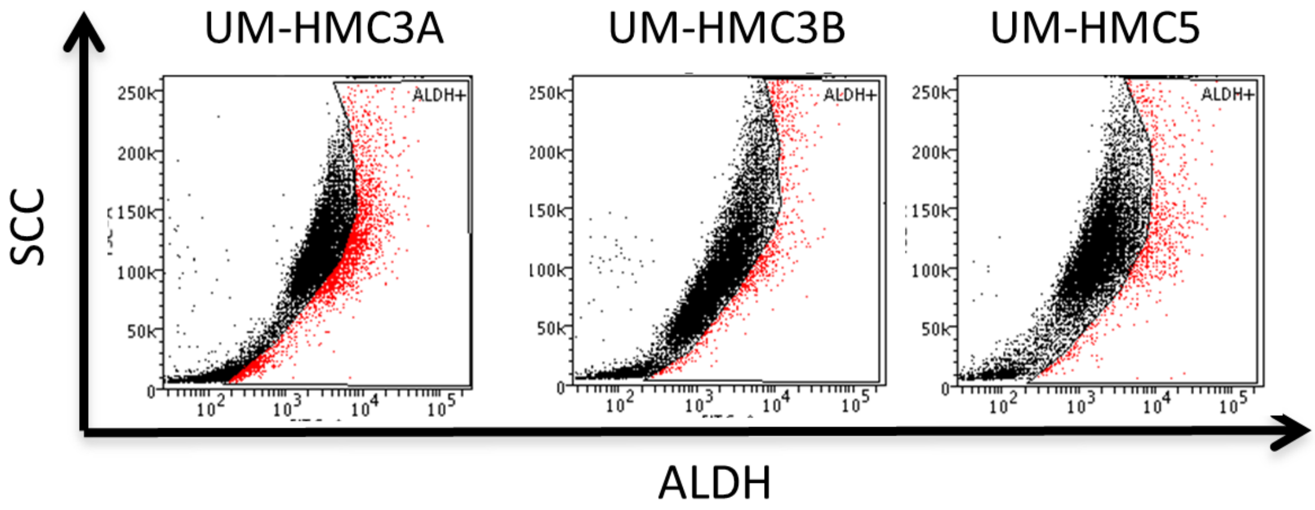

Supplementary Figure S2: Presence of ALDH⁺ cells in MEC cell lines. Detection of ALDH activity in UM-HMC3A, UM-HMC3B, and UM-HMC5 (red) by flow cytometry.

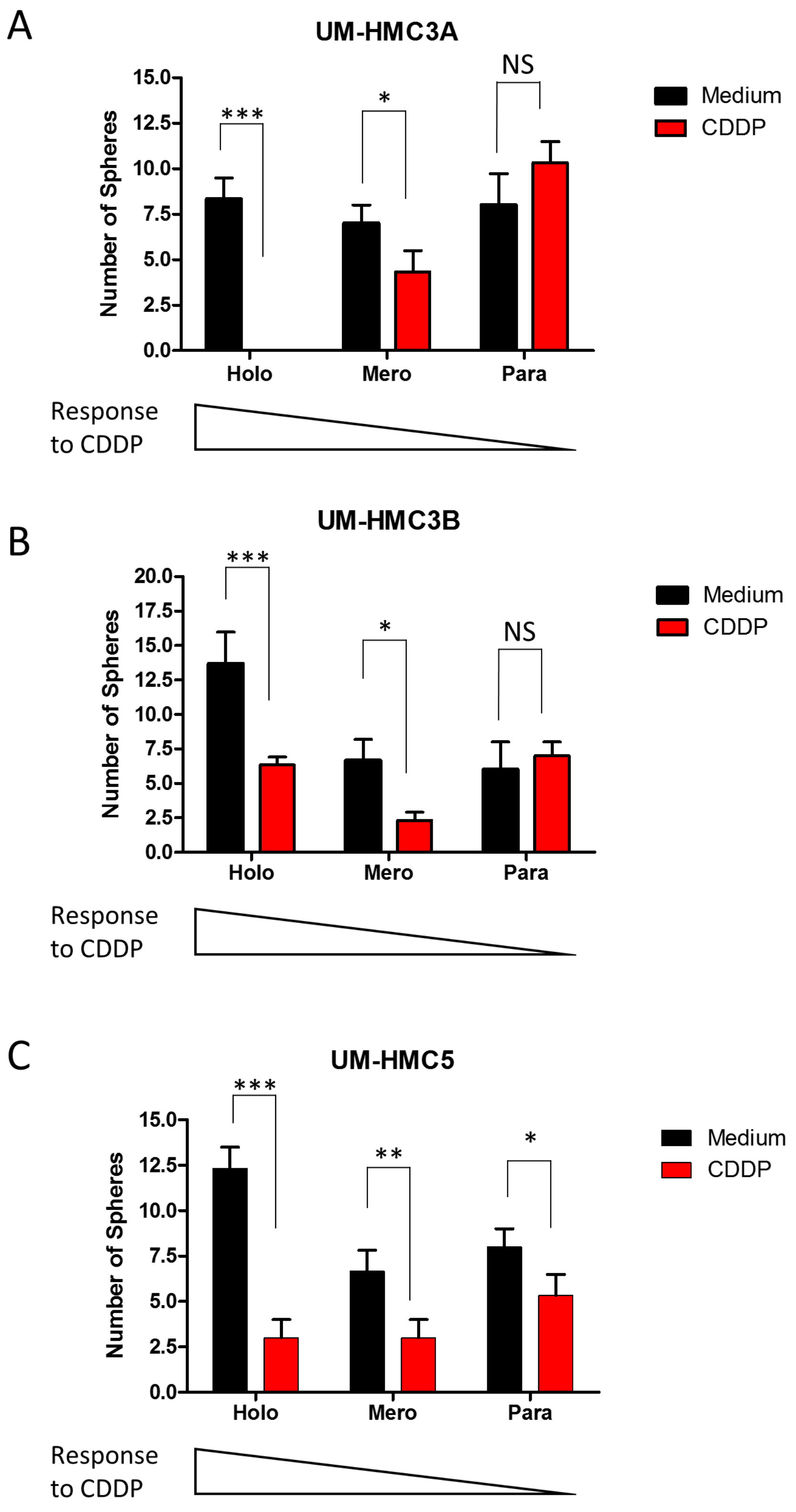

Supplementary Figure S3: Cisplatin induces accumulation of a subpopulation of tumor spheres. A. Cisplatin alone reduces the number of holospheres and merospheres, but not paraspheres, from UM-HMC3A (NS $p>0.05$; *$p<0.05$; ***$p<0.001$). **B.** Similar to UM-HMC3A, paraclones from UM-HMC3B do not respond to Cisplatin (NS $p>0.05$; *$p<0.05$; ***$p<0.001$). **C.** Cisplatin significantly reduces the number of holospheres, merospheres, and paraspheres in UM-HMC5 (*$p<0.05$; **$p<0.005$; ***$p<0.001$).

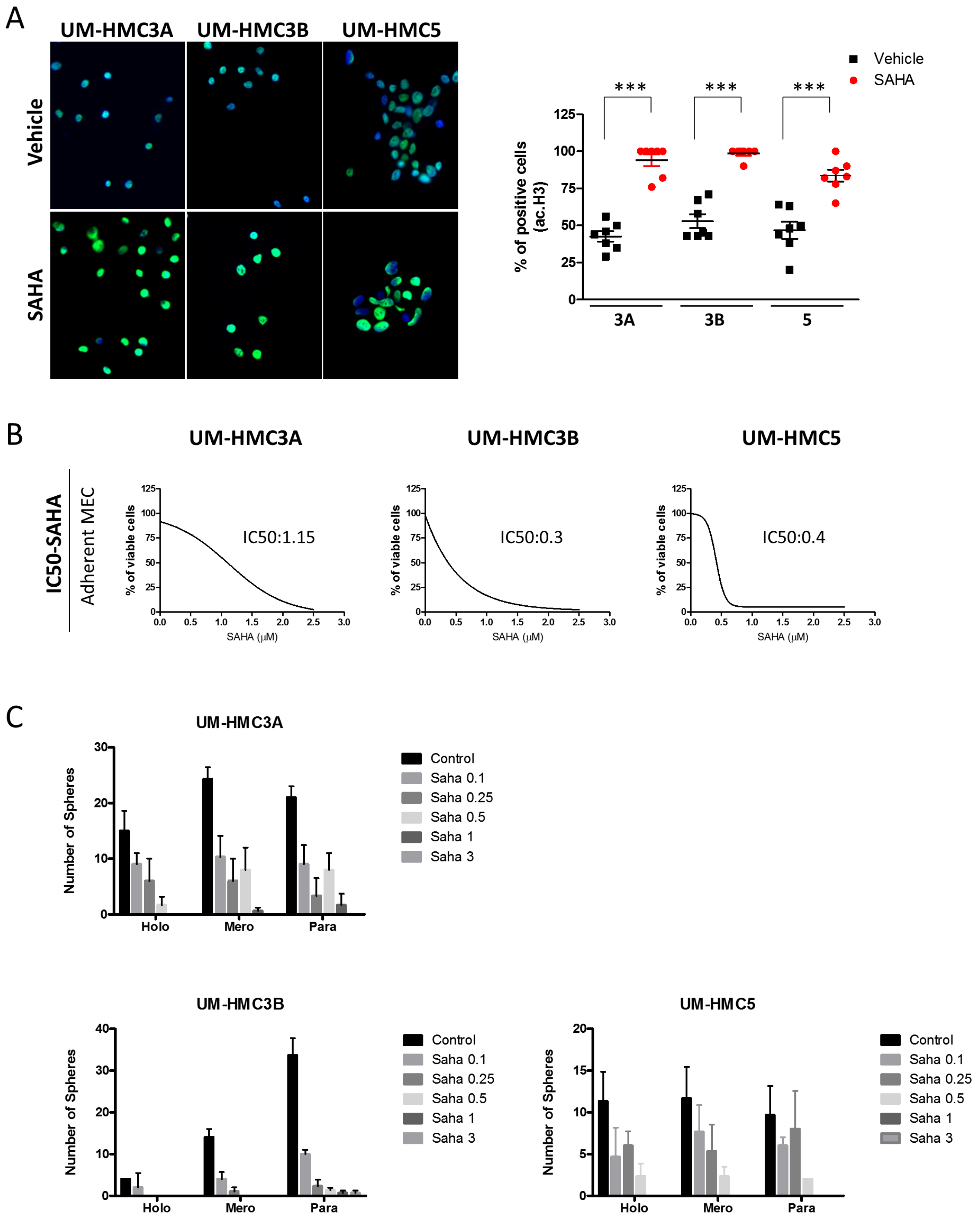

Supplementary Figure S4: SAHA reduces CSCs in MEC cell lines. A. Tumor cells undergoing histone acetylation (ac.H3) upon administration of SAHA (***p<0.001). **B.** Determination of the IC_{50} of SAHA using a cell viability assay. **C.** Total number of tumor spheres derived from UM-HMC3A, UM-HMC3B, and UM-HMC5 receiving different concentrations of SAHA.

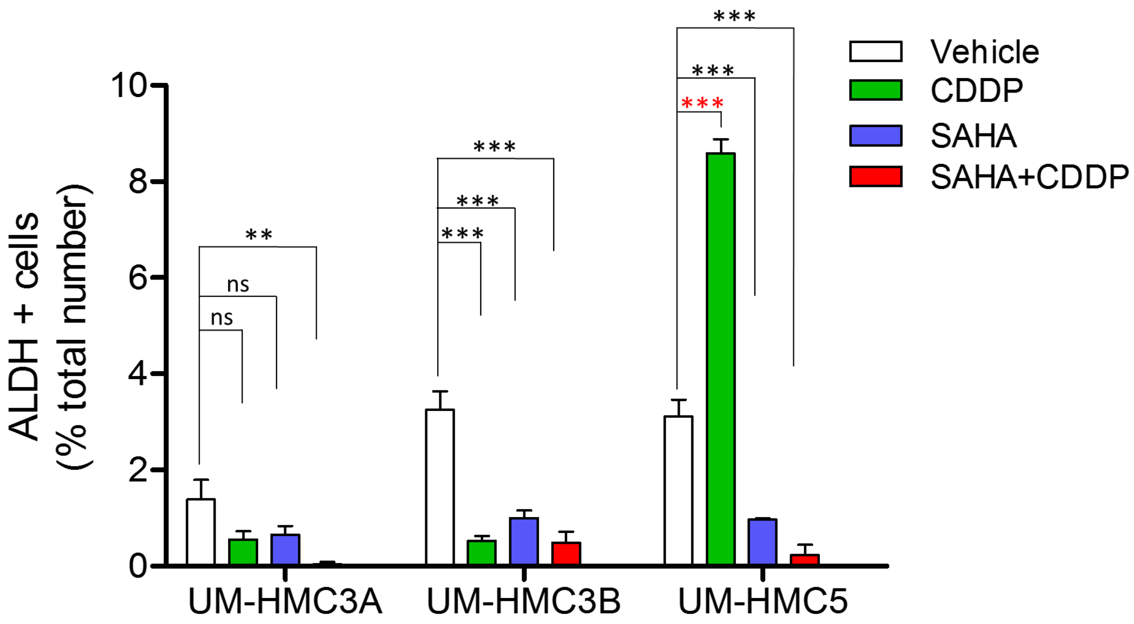

Supplementary Figure S5: The impact of the two hit approach, SAHA followed by Cisplatin, on CSCs. The percentage of tumor cells positive for ALDH activity in response to vehicle (white), Cisplatin (green), SAHA (blue), and SAHA plus Cisplatin (red).
